# Assessing the adoption of the FAIR principles in Italian environmental research infrastructures

**DOI:** 10.1016/j.patter.2025.101420

**Published:** 2025-11-12

**Authors:** Enrica Nestola, Gregorio Sgrigna, Gianmarco Ingrosso, Andrea Tarallo, Davide Raho, Cristina Di Muri, Alexandra Nicoleta Muresan, Ilaria Rosati

**Affiliations:** 1 Research Institute on Terrestrial Ecosystems (IRET), National Research Council of Italy (CNR), Campus Ecotekne, Via Monteroni 165, 73100 Lecce, Italy; 2 National Biodiversity Future Center (NBFC), Piazza Marina 61, 90133 Palermo, Italy

**Keywords:** environmental research infrastructures, data interoperability, FAIR strategies, FAIR choices, FAIR practices, FAIR implementation technologies, digital object management, FAIR convergence, FAIR communities, FAIR challenges

## Abstract

This study investigates the adoption of the FAIR (Findable, Accessible, Interoperable, and Reusable) principles by 14 environmental research infrastructures (RIs) operating at the Italian level. Through a three-step process (surveys, interviews, and a resource analysis), we explore the diverse FAIR practices adopted across four environmental subdomains, namely atmosphere, marine, biosphere, and geosphere. The findings reveal significant heterogeneity in the implemented practices, with ongoing efforts to converge on common strategies, particularly in the marine subdomain. Serving as a stepping stone toward more coordinated FAIR implementations, the analysis herein provides a solid foundation for monitoring future progress regarding the adoption of FAIR practices across environmental RIs within and beyond Italy and Europe.

## Highlights

• Assessing how environmental research infrastructures apply FAIR principles in Italy
• Adopted FAIR implementation practices are very heterogeneous
• Key reference for tracking future progress in adopting FAIR practices
• Strategic insights for the design and development of the ITINERIS HUB

## The bigger picture

Addressing today’s environmental crises requires not only generating new scientific knowledge but also ensuring that both existing and emerging knowledge can be effectively shared and reused by others. Environmental research infrastructures (RIs) are central to this effort. They provide the frameworks and resources needed to collect, preserve, and share digital scientific outputs over the long term, making them available for evidence-based policy.

The FAIR (Findable, Accessible, Interoperable, and Reusable) principles have emerged as a transformative way to guide the management of digital resources. By implementing these principles, RIs strengthen the value of environmental data and foster more coordinated digital ecosystems, which are essential for tackling current global challenges such as climate change, biodiversity loss, and natural hazards. Yet, putting FAIR principles into practice is not straightforward. Different scientific communities face diverse needs, and their choices about how to apply FAIR principles are often uneven or not consistently documented. This study provides a detailed overview of how environmental RIs in Italy are implementing the FAIR principles. By identifying shared practices and challenges, this work represents a starting point for tracking how FAIR adoption evolves and for guiding other communities in building their own FAIR-compliant platforms and services. These findings are relevant beyond the environmental domain and offer practical insights for the development of more coordinated and reusable digital ecosystems across science.

## Graphical abstract

**Figure:**
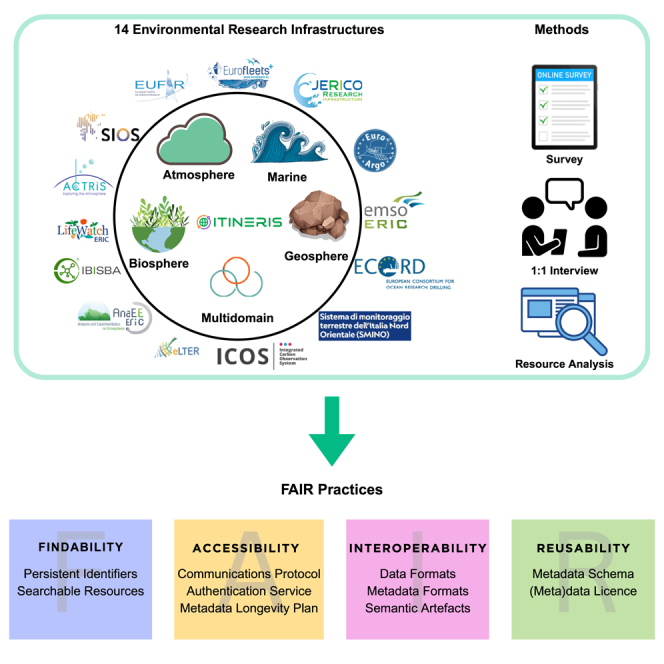

## Teaser

The authors analyze how 14 environmental research infrastructures (RIs) implement the FAIR (Findable, Accessible, Interoperable, and Reusable) principles in Italy. The study uncovers diverse practices across the atmosphere, marine, biosphere, and geosphere subdomains. Results reveal not only strong heterogeneity but also emerging convergence, particularly in the marine subdomain. By highlighting transferable FAIR strategies, this work can guide other scientific communities interested in FAIR compliance and support the development of new digital ecosystems within and beyond Italy.

## Introduction

Addressing global environmental challenges such as climate change, pollution, biodiversity loss, and land degradation demands efficient data management and sharing strategies across multiple scientific domains.^1^^,^^2^^,^^3^ FAIR principles and open science practices are the two key pillars advocated by the European Commission for research and innovation programs. Together, they can accelerate the pace of scientific discovery and enable solutions for global crises.^4^^,^^5^^,^^6^ The FAIR principles, “FAIR” representing an acronym for “Findable, Accessible, Interoperable, and Reusable,” have been acknowledged as a revolutionary framework for guiding the integration and reuse of research results. Since their introduction in 2016, the FAIR principles have had an extraordinary impact on multiple research areas, with the commentary paper of Wilkinson et al.^7^ reaching almost 17,000 citations in just 9 years, thus becoming a cornerstone of the international scientific community^8^ (Google Scholar, accessed on May 9, 2025). In the wake of this success, a significant number of international projects and initiatives have worked on developing practical solutions that facilitate the application of FAIR principles in scientific research, such as ENVRI-FAIR,^9^ GO FAIR,^10^ FAIRsFAIR,^11^ FAIR IMPACT,^12^ and ENVRI-Hub NEXT.^13^ One of the strengths of the FAIR principles is that they implicitly acknowledge diverse and valid implementation choices, ensuring a degree of flexibility to accommodate the needs of specific communities. This strength, however, comes with both advantages and drawbacks, including broad applicability but also potential ambiguity.^14^ In fact, while the FAIR principles provide crucial guidance about “where” we need to go, finding practical solutions for “how” to implement them remains a critical step.^15^ As clearly conveyed by Wittenburg et al.,^16^ the scientific community needs specific implementation practices that can satisfy the FAIR principles in different research domains. This is supported by the observation that 80% of studies referencing the initial FAIR publication specifically focus on practical implementations.^17^ In fact, alongside the “political hype” generated by the acronym, an increasing number of organizations and communities are actively striving to implement technical choices that align with the FAIR guiding principles.^18^ Taking all necessary steps in this direction requires considerable resources and effort since it involves a general change in the practices of all the actors involved. Importantly, the urgency of FAIR compliance goes beyond concerns about data reproducibility or scientific misconduct; it also relates to the potential scientific innovations that FAIR data can foster for addressing global societal challenges.^19^^,^^20^^,^^21^ In this context, research infrastructures (RIs) have the responsibility to bridge the gap between the FAIR principles and the practical approaches needed to achieve FAIR compliance.^22^^,^^23^

Europe has a rich landscape of RIs, which are defined as facilities that provide resources and services for research communities to conduct top-level research and drive innovation in their fields.^24^ Different types of RIs are typically classified as physical, digital (or virtual), and hybrid infrastructures, depending on the nature of the access and resources they offer. A physical RI is a facility that provides physical access to scientific equipment (e.g., observatories, laboratories, and instruments) to foster scientific research and innovation. Researchers use these facilities to conduct experiments, collect data, and share knowledge across a wide range of disciplines. In contrast, a digital RI offers online tools, services, and data to support scientific research without requiring the physical presence of the user. These infrastructures allow researchers to access and analyze data, collaborate virtually, and use distributed computing resources across different institutions and countries. Finally, many RIs operate as hybrid infrastructures, combining both physical and digital components to support a broader spectrum of scientific activities.

In all their forms, RIs play a fundamental role in providing the high-quality information and services needed to find actionable strategies for addressing the complex environmental issues faced by our society.^25^ In Europe, RIs are guided by the European Strategy Forum on Research Infrastructures (ESFRI), a strategic body that coordinates policymaking and supports the implementation and continuous monitoring of RIs.^26^ ESFRI RIs are actively engaged in the European Open Science Cloud (EOSC) through the shared goal of fostering open and FAIR research data and services. Accordingly, a specific ESFRI-EOSC task force^27^ was established in June 2023 to align collective interests and maximize efforts toward the integration of RIs within the open, multidisciplinary environment that EOSC envisions for sharing and reusing data, tools, and services.^28^

The connection between ESFRI RIs and the EOSC builds upon earlier efforts, such as the establishment of five Science Cluster projects in 2019 within the H2020 framework program.^29^ Among these, the cluster of the European Environmental RIs (ENVRI Cluster; see ENVRI.eu) focuses on atmospheric, marine, solid earth, ecosystem, and biodiversity research and includes RIs that provide research products and services from these key areas of the earth system.^30^ Building on this, the project “Italian Integrated Environmental Research Infrastructures System (ITINERIS)” emerged as a national effort to move in the direction outlined by the ENVRI and EOSC frameworks.^31^ The ITINERIS coordinates a network of national nodes from 22 RIs, comprising physical, digital, and hybrid infrastructures. The project is aimed at establishing the Italian hub of RIs in the environmental scientific domain, providing access to data and services for addressing the current and expected environmental challenges (i.e., ITINERIS HUB).^32^ The ITINERIS project is also committed to enabling federated access to FAIR research data and services of national RIs, facilitating connections with the EOSC at the European level, and offering guidance on standard FAIR approaches for emerging national RIs.

In light of all that has been discussed thus far, it is clear that (1) FAIR principles are recognized by the European Commission as fundamental building blocks of the EOSC, (2) RIs support science and innovation by following an open science framework in accordance with the FAIR principles, and (3) the application of the FAIR principles depends on the specific needs of the scientific community. Consequently, analyzing the FAIR solutions adopted by RIs is critical, as it provides a basis for monitoring future progress in this direction. However, to our knowledge, a systematic investigation of how the FAIR principles are being adopted across environmental RIs is lacking. This study is aimed at providing a structured analysis of the FAIR practices implemented by 14 environmental RIs at the national Italian level, encompassing multiple environmental subdomains.

The impact of our analysis can be considered 3-fold. First, it promotes the transfer of FAIR awareness to emerging and fast-developing RIs, thus strengthening the national RI landscape in the environmental field and the convergence of RIs toward common practices. Second, it provides a basis for monitoring the ongoing adoption of FAIR practices by environmental RIs and tracking their future advancements. Finally, it offers essential insights that can inform and guide the development of the national hub envisioned in the context of the ITINERIS project.

## Results

**Figure 1:**
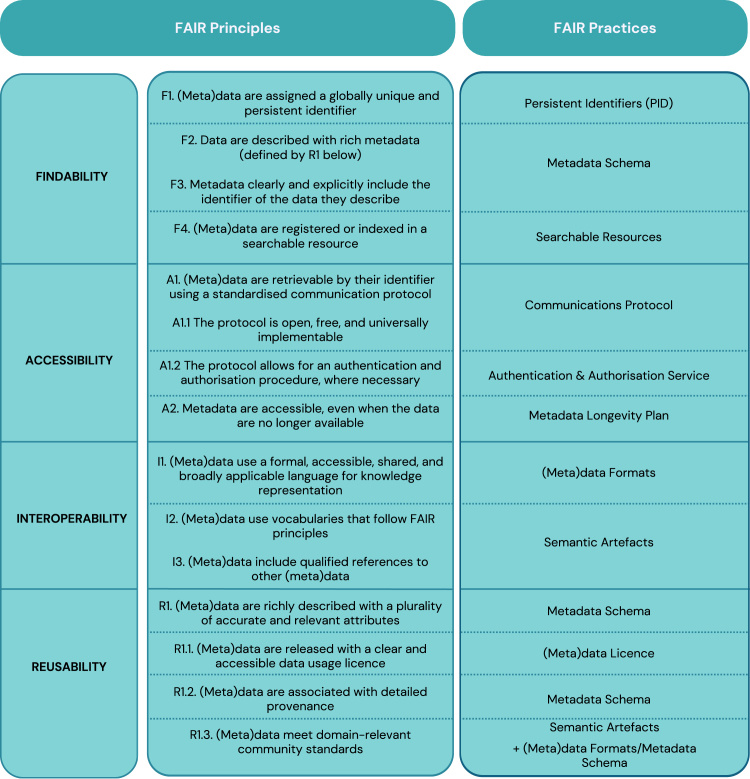
From FAIR principles to FAIR practices The 4 FAIR principles and their 15 subprinciples as reported in the GO FAIR initiative (go-fair.org), together with the corresponding FAIR practices analyzed within this study.

The FAIR practices adopted by 14 environmental RIs involved in the ITINERIS project are presented below, together with their related data sources.^33^ Nine FAIR practices associated with 15 FAIR subprinciples were identified (Figure 1). The results are organized into four subsections according to the four FAIR principles: Findability, Accessibility, Interoperability, and Reusability. Each subsection begins with a brief description of the FAIR practice and its connection to the relevant subprinciples (Figure 1).

### Findability

Three FAIR practices were identified for implementing the four subprinciples of Findability (F1–F4; Figure 1): persistent identifiers (PIDs), metadata schemas, and the identification of searchable resources.

#### Persistent identifiers

PIDs are defined as unique, machine-readable codes that are assigned to digital resources to make them easily discoverable and able to be referenced.^34^^,^^35^ Subprinciple F1 requires that each digital object (DO) has been assigned a globally unique and persistent identifier, where “globally unique” means it unambiguously refers to a single resource and “persistent” means that the identifier continues to reference the same resource, even if it is relocated or no longer exists.^15^ PIDs must also have web accessibility/resolvability via services that redirect users to the identified resource or its metadata.^36^ In this section, we explore which identifier services were used within the analyzed RIs.

**Figure 2:**
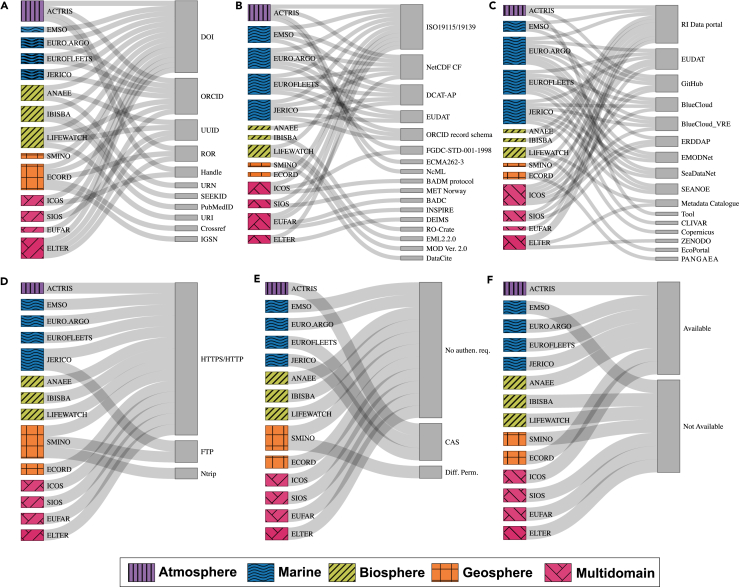
Sankey diagrams representing the FAIR implementation practices adopted by 14 research infrastructures for the Findability and Accessibility principles (A–F) The diagrams show the (A) persistent identifiers, (B) metadata schemas, (C) searchable resources, (D) communication protocols, (E) authentication and authorization services, and (F) metadata longevity plans.

Overall, a total of 11 different PIDs were listed. However, all 14 RIs exhibited unanimity in their use of digital object identifiers (DOIs), often combined with other PIDs, to uniquely identify and resolve DOs (Figure 2A). The second-most-common PID used to identify DOs was the universally unique identifier (UUID), which was adopted by four infrastructures (Figure 2A). The combination of multiple PIDs in some RIs arose from the need to manage various types of DOs and suggests an effort to enhance the identification of different resources. For instance, the Open Researcher and Contributor ID (ORCID) was widely adopted by RIs for the identification of individuals, the Research Organization Registry (ROR) was used for institutional affiliations, Crossref was employed for research funders, and International Generic Sample Numbers (IGSNs) were utilized for physical sample identification and tracking purposes. In addition, the adoption of multiple PIDs reflects that identifiers can be assigned at either the DO or the metadata level.

#### Metadata schemas

A metadata schema consists of a predefined set of elements that is used to structure metadata, specifying element names, meanings, and content rules. When machine readable, it enables information to be described and managed in a structured and interoperable way.^37^ The F2 subprinciple indicates the importance of describing DOs with rich metadata, whereas the F3 subprinciple requires metadata to clearly include the identifier of the described DO. This section presents an analysis of the metadata schemas employed by the RIs for describing their resources.

A total of 17 different metadata schemas were identified (Figure 2B). Among these, the International Organization for Standardization (ISO) schemas were by far the most widely used, with 11 RIs adopting ISO19115/19139. Notably, the NetCDF Climate and Forecast (CF) metadata conventions were widely employed within the atmosphere, marine, and multidomain RIs, suggesting a convergent pattern across these communities. Beyond the ISO schemas and NetCDF, 15 other distinct metadata schemas were employed by the RIs, and this variety is explained by the diversity of the resources described. For example, Metadata for Ontology Description and Publication (MOD) is a metadata schema that was designed for semantic artifacts (SAs),^38^ the ORCID record schema is used for researchers,^39^ and the Data Catalog Vocabulary Application Profile (DCAT-AP) is a metadata schema used for European data portals.^40^

#### Searchable resources

Searchable resources are those services that allow DOs to be found.^41^ In accordance with subprinciple F4, DOs must be indexed in a way that makes them easily findable and accessible, typically by storing or registering them in a public resource such as a data archive or repository.^42^ Ensuring that public data remain accessible in repositories is essential for advancing science, with additional benefits for strengthening democracy and promoting justice.^43^ In this section, for each RI, we analyze where the DOs are registered or indexed. We considered structured platforms such as institutional repositories, registries, (meta)data catalogs, archives, and all the other systems that make DOs findable.

There was a clear tendency among the RIs to use their own dedicated portals and catalogs to make their DOs findable and accessible. Indeed, 11 RIs chose this option as their primary means of dissemination (see the RI Data Portal and the RI Metadata Catalogue, Figure 2C). However, it is also important to highlight the fact that RIs simultaneously use other external services for the management and storage of their DOs. Examples of these services are collaborative data infrastructures or servers such as the European Data Infrastructure (EUDAT, https://www.eudat.eu), which is used for different scientific disciplines (six RIs) and the Environmental Research Division’s Data Access Program (ERDDAP, https://github.com/ERDDAP/erddap), which is used for more specific research areas (e.g., oceanographic data; three RIs). Another interesting result that emerged from our analysis is that all marine RIs converged toward common solutions, such as SeaDataNet (https://www.seadatanet.org), SEANOE (https://www.seanoe.org/), BlueCloud (https://blu-cloud.org/), EUDAT, ERDDAP, and the European Marine Observation and Data Network (EMODNet, https://emodnet.ec.europa.eu/en). This indicates strategic coherence within the domain and a consistent approach for making marine data discoverable through the same channels.^44^ Finally, GitHub emerged as the collaborative software development platform where RIs store and share their codes and related documentation (Figure 2C).

### Accessibility

Three practices were identified to implement the four subprinciples of Accessibility (A1, A1.1, A1.2, and A2; Figure 1): communication protocols, authentication and authorization services, and metadata longevity plans.

#### Communication protocols

Standardized communication protocols are defined as a “set of rules and conventions that dictate how data is exchanged between devices or systems in a network.”^45^ Applied to the context of RIs, communication protocols define the rules, conventions, and procedures that govern the exchange of DOs between information systems such as client applications (users) and the web servers of RIs, ensuring accurate and reliable data transfer processes. The Accessibility principle encourages free and open-source communication protocols (A1.1) to facilitate the retrieval and reuse of DOs (A.1). In fact, the choice of protocol can significantly impact the transmission of information and provide enhanced data security.^15^

Various methods for accessing DOs were outlined during the analysis of the RIs (Figure 2D). In terms of communication and transmission protocols, all the analyzed RIs relied on the hypertext transfer protocol secure (HTTPS) (Figure 2D). SMINO and JERICO also support the file transfer protocol (FTP) in addition to HTTP/HTTPS (Figure 2D). Additionally, SMINO uniquely supports the Networked Transport of RTCM via Internet Protocol (Ntrip), a protocol that was specifically designed for streaming Global Navigation Satellite System (GNSS) correction data in real time^46^ (Figure 2D). In summary, while HTTPS/HTTP represents the universal foundation for implementing web-based communication across all RIs, the selective use of protocols such as FTP and Ntrip underscores specific domain requirements despite certain trade-offs in terms of security or technological currency.

In addition to the communication protocol analysis, we also examined the methods, services, and tools that are available for accessing DOs within the analyzed RIs. While the underlying protocols (e.g., HTTP/HTTPS) handle data transmission processes, it is through access interfaces (such as REST application programming interfaces [APIs] and SPARQL Protocol and RDF Query Language [SPARQL] endpoints) that users effectively interact with and retrieve structured information. More specifically, APIs permit machine-to-machine communication, offering advanced features such as querying, subsetting, and update checking, providing significant advantages over mere bulk file downloads.^47^ As a result, some RIs have developed dedicated APIs for the harvesting of data and metadata. Eight of the same RIs that use HTTPS/HTTP also support REST API technology. These include RIs operating in different subdomains, such as ACTRIS, Euro-Argo, EMSO, Eurofleets, JERICO, eLTER, LifeWatch, SMINO, and ECORD. A smaller number of RIs provide access to their DOs through the SPARQL endpoints, which enable users to perform complex, semantic queries on RDF-structured data.^48^ Among the analyzed infrastructures, LifeWatch, ICOS, Eurofleets, and JERICO offer this type of access.

#### Authentication and authorization services

Authentication is the process of proving that the user with a digital identity who is requesting access is the rightful owner of that identity, while authorization consists of evaluating the access type or access rights a digital identity should have within an environment.^49^ Subprinciple A1.2 implicitly highlights the fact that FAIR does not mean “open.” In fact, even DOs with high levels of protection and confidentiality can still adhere to the FAIR principles.^50^ When a DO has restrictions, the associated access procedure requires extra steps, such as confirming the identity of the individual requesting access (authentication) and verifying that the user’s profile satisfies the conditions for accessing the DO (authorization). A requester can be either a human or a machine agent.^15^

Most of the observed RIs offer full open access to their research products, and few of them require a mandatory authentication step (e.g., ACTRIS, Eurofleets, and JERICO) through the Central Authentication Service (CAS) before downloading the DOs (Figure 2E). In all RIs, DO uploading and the use of eventual additional analytical tools are instead allowed only after an authentication step. This addresses the requirement for RIs to manage user access and privileges effectively, distinguishing between novice and proficient users or different roles.

#### Metadata longevity plans

A metadata longevity plan ensures that metadata remain accessible even when the DOs that they describe are not (A2). Ideally, this point should be addressed within the data management plan (DMP) of each RI to implement specific strategies for the long-term management and preservation of metadata.^51^

In our analysis, 7 out of 14 RIs provided explicit information about the strategies adopted for the management and preservation of metadata (Figure 2F). These results reflect a growing awareness of the importance of preservation strategies, yet their actual implementations remain limited.^52^

### Interoperability

Two FAIR practices were analyzed for the implementation of three Interoperability subprinciples (I1, I2, and I3; Figure 1): (meta)data formats and SAs.

#### (Meta)data formats

To facilitate the exchange of information among systems, both data and metadata should be structured using standard formats^53^ (I1, Figure 1). The knowledge representation languages mentioned in I1 are formal systems that are used to represent and encode knowledge in a structured and machine-readable manner. In terms of syntactic interoperability, it means that (meta)data are provided in a common and shared format that can be read by machines as well. The format and extension are dependent on the type of corresponding DO. In this section, we analyze the formats used within the RIs for uploading, downloading, or machine access to both data and metadata.

**Figure 3:**
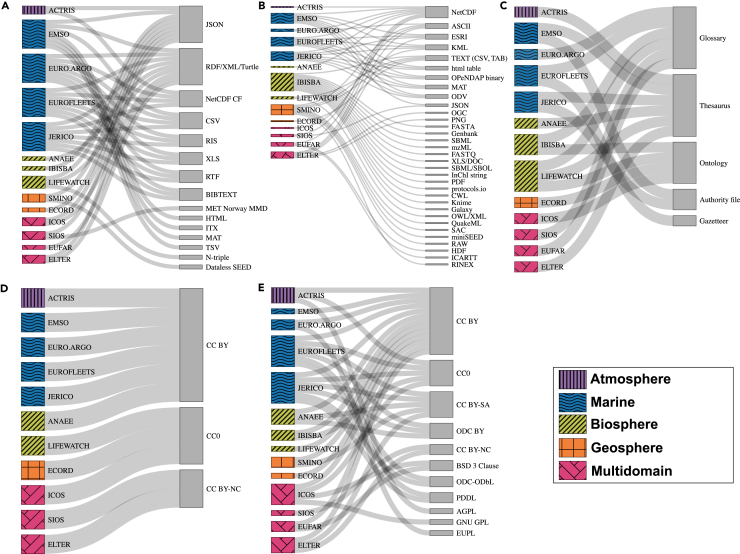
Sankey diagrams representing the FAIR implementation practices adopted by 14 research infrastructures for the Interoperability and Reusability principles (A–E) The diagrams show the (A) metadata formats, (B) data formats, (C) semantic artifacts, (D) licenses associated with metadata, and (E) licenses associated with other DOs.

A considerable variety of ways to make metadata information available were identified, with 15 different format types observed. Among these, the JavaScript Object Notation (JSON) and the “RDF/XML/Turtle” (i.e., Resource Description Framework/eXtensible Markup Language/Terse RDF Triple Language) groups stood out as prominent choices (Figure 3A). These two formats were selected by nine RIs and are sometimes used concurrently (e.g., ICOS, EMSO, eLTER, and LifeWatch), while other times they are used alternatively: JSON is used in ACTRIS, SIOS, IBISBA, SMINO, and ECORD, whereas RDF/XML/Turtle is used by Euro-Argo, Eurofleets, EUFAR, JERICO, and AnaEE. However, the JSON format is represented across all the subdomains in the analysis, whereas the RDF/XML/Turtle group is not represented in the atmosphere and geosphere subdomains.

Among all the FAIR practices observed in our analysis, the data format is the one that registered the highest number of categories, with 33 distinct data formats observed (Figure 3B). These results underscore the fundamental importance of data collection and data sharing at the core of most RIs. In some environmental subdomains, data collection can cover a wide range of types, leading to differences in the formats in which they are expressed. This large number is due primarily to the influences of two RIs, IBISBA and SMINO, which belong to the biosphere and geosphere subdomains, respectively. These two RIs alone contribute 18 exclusive format types (not shared with other RIs). The most widely used format is NetCDF, adopted by nine RIs. No other data format stands out significantly. However, when considering the ASCII and TEXT categories together with NetCDF, they cover all 14 RIs.

From a subdomain perspective (Figure 3B), the greatest variety of data formats is observed within the biosphere and marine subdomains. However, while the biosphere (as highlighted above with IBISBA) shows high heterogeneity with no clear convergence to specific formats, the marine domain exhibits diversity with a notable overlap in the formats adopted (e.g., NetCDF and ESRI).

#### Semantic artifacts

Principle I2 encourages the use of common and shared “vocabularies” to represent the contents and meanings of DOs. Rather than using the general umbrella term “vocabulary,” in this study, we used the most recent and community-accepted term of “semantic artifact,” in line with Di Muri et al.^54^ and Le Franc et al.^55^ SAs (e.g., controlled vocabularies, ontologies, thesauri, etc.) are machine-readable and machine-actionable formalizations of concepts that can be used and exchanged to encode and predictably decode information, thereby enabling the discovery, integration, and reuse of information by both humans and machines.^55^ Furthermore, through SAs, meaningful connections between (meta)data can be established, as described by subprinciple I3. This means that (meta)data should include qualified references to other associated (meta)data whereby the nature of these references is also clearly specified. Such references enrich the contextual knowledge of DOs by providing an interlinked network of (meta)data.^15^ For instance, ontologies specify a wide range of precisely defined relationships, namely object properties, that act as predicates for defining these qualified references between two entities (i.e., a subject and an object).^15^ For this reason, we analyzed the SAs used by the RIs to assess the FAIR practices implemented to address principles I2 and I3 (Figure 1).

In this study, the SAs were grouped into five main categories following the classification scheme described in Di Muri et al.^54^ The identified categories are listed here based on an increasing level of complexity: glossaries, gazetteers, authority files, thesauri, and ontologies (Figure 3C). Thesauri and glossaries are the most commonly used types of SAs and are adopted by six RIs, whereas ontologies are employed by four RIs (Figure 3C). The majority of the SAs used by the analyzed RIs are available in semantic catalogs such as the NERC Vocabulary Server,^56^ EMBL-EBI Ontology Lookup Service,^57^ and EcoPortal,^58^ whereby the latter is managed by the LifeWatch RI. While most RIs use existing SAs, five RIs (i.e., ACTRIS, ICOS, LifeWatch, eLTER, and AnaEE) are also SA creators and managers.

### Reusability

Four FAIR practices were identified for describing four reusability subprinciples (R1, R1.1, R1.2, and R1.3): metadata schemas, (meta)data formats, SAs, and (meta)data licenses (Figure 1). Given that practices related to metadata schemas, metadata formats, and SAs have already been described, this section focuses solely on the usage licenses adopted by the analyzed RIs.

#### Licenses

Accompanying DOs with explicit and readily accessible usage licenses (R1.1) are essential for providing a legal framework that delineates the terms and conditions governing the utilization of the DOs. This ensures clarity about which actions are permitted or restricted.^59^ A clear and accessible license is crucial for enabling not only humans but also automated systems to ascertain the permissible uses of DOs, thus facilitating compliance and responsible reuse. For example, the lack of clearly available usage licenses has been identified as a significant barrier to the reuse of SAs.^60^

The most common licenses adopted by RIs operating in the environmental domain were open Creative Commons licenses (https://creativecommons.org/), both for metadata (Figure 3D) and for other DOs (Figure 3E). Creative Commons licenses offer a standardized way to provide public permission to use copyrighted works while ensuring the recognition of the original creator.^61^ Eleven out of the 14 analyzed RIs (ACTRIS, EMSO, Euro-Argo, Eurofleets, JERICO, AnaEE, LifeWatch, ECORD, ICOS, SIOS, and eLTER) use Creative Common licenses for their metadata (Figure 3D). In our analysis, the licenses identified for metadata were Creative Commons Attribution (CC BY), Creative Commons Attribution-NonCommercial (CC BY-NC), and Creative Commons Zero (CC0) (Figure 3D). These licenses specify the conditions under which metadata can be reused: CC BY allows reuse with appropriate credit given to the original source, CC BY-NC permits reuse for non-commercial purposes only (still requiring attribution), and CC0 allows for unrestricted reuse without the need for attribution.

With respect to the other DOs, the main license types identified among the Creative Commons suite were CC BY, followed by CC0, Creative Commons Attribution-ShareAlike (CC BY-SA), and CC BY-NC (Figure 3E). The CC BY-SA license allows the reuse of the underlying material, requiring that any adaptations be shared under the same license terms. Among other types of DO licenses, Open Data Commons (ODC) licenses (https://opendatacommons.org/) are designed specifically for open data rather than for different types of content.^61^ Within the umbrella of ODC, we identified three main license types, the Public Domain Dedication and Licence (PDDL), the Attribution Licence (ODC-By), and the Open Database Licence (ODC-ODbL), which are used by Eurofleets, JERICO, and AnaEE (Figure 3E). Finally, open source licenses, such as the Berkeley Source Distribution (BSD) license, GNU General Public License (GPL), GNU Affero General Public License (AGPL), and European Union Public Licence (EUPL), are conventionally used for software/workflows^62^ (Figure 3E).

## Discussion

The discussion is structured into four subsections corresponding to each of the four FAIR principles and a final subsection that addresses specific FAIR challenges in the environmental domain.

### Findability

Our analysis indicated that all 14 RIs uniformly use DOIs to support findability (Figure 2A). This finding aligns well with prior evidence. In fact, Peterseil et al.^63^ noted that 90% of the RIs in the ENVRI-FAIR project preferentially used DOIs. DOIs offer stability through a well-established infrastructure (i.e., the International DOI Foundation maintains the DOI system^64^) and can be assigned to various DOs. Although maintaining DOIs can be costly without a solid financial plan, our results showed that there is a strong consensus on referring to a third party to generate an identifier that guarantees longevity and project independence. Unlike DOIs, PIDs based on UUIDs (e.g., uniform resource identifier [URI]) are free of charge, but their persistence depends on the longevity of the infrastructure that provides them. On the other hand, systems such as uniform resource names (URNs) benefit from the support of national libraries.^65^

A consistent pattern also emerged in the metadata schema analysis, which revealed strong alignment across RIs, with ISO 19115/19139 as the leading standard, complemented by widespread use of the NetCDF conventions (Figure 2B). The ISO 19115/19139 metadata standard is widely adopted in environmental RIs because of its international recognition, robust support for spatial and temporal metadata elements, and flexibility for extension or profiling to meet domain-specific needs.^66^ Similarly, the broad adoption of NetCDF CF as a metadata convention has numerous advantages.^67^ Among these, the most significant benefits are its widespread usage and compatibility: CF conventions are extensively employed, are supported by numerous tools, and ensure backward compatibility, facilitating data sharing, analysis, and reusability.^68^^,^^69^ According to Leipzig et al.,^70^ in recent decades, many initiatives have developed and sustained a wide range of metadata schemas, each with its own purpose. While there might be some convergence among different schemas, a concerted effort to map and align the varying terminologies—or at least a core set of terms—across these schemas is needed, ensuring their interoperability in terms of structures and semantics.^71^ In this context, the general adoption of DCAT-AP reflects ongoing efforts in this direction, as it serves as the common metadata schema used to describe the European data portals in a straightforward, standardized, and comprehensive manner.^40^

The choice to rely on their own dedicated portals and catalogs to manage the discovery and access of their DOs is a common feature among the analyzed RIs (Figure 2C). These systems, managed by each RI, are likely to provide tailored interfaces and search functionalities for meeting the specific needs of their user communities. On the other hand, the additional adoption of broader discovery portals, such as the B2FIND metadata indexing service of EUDAT (https://b2find.eudat.eu/), by several RIs shows a growing recognition of the benefits of harvesting resources on platforms with wider reach. Notably, EUDAT serves a diverse range of scientific domains, suggesting that multidisciplinary discovery portals are becoming increasingly important for promoting data accessibility.^72^^,^^73^

### Accessibility

With respect to the standardized communication protocol, all analyzed RIs use HTTPS (Figure 2D). This protocol is widely adopted across web-based services because of its standardization and ease of implementation. In particular, HTTPS offers encrypted communications, ensuring the confidentiality and integrity of DOs in transit.^74^ Used only by JERICO and SMINO (Figure 2D), the FTP is one of the earliest-defined communication protocols and has undergone an extensive evolution process since its original specification.^75^ It remains in use today, particularly for bulk file transfers, due to its efficiency in handling large DOs. However, in terms of machine-to-machine accessibility services, FTP links fall short in addressing the complex needs of globally distributed scientific and operational requirements.^76^ The exclusive use of the Ntrip protocol by SMINO (Figure 2D) reflects the focus of this infrastructure on seismological data services. In fact, this protocol is widely used in real-time kinematic (RTK) positioning applications, and it is valuable in contexts where highly precise geolocation data are needed.^77^ Moreover, the inclusion of SPARQL endpoints in LifeWatch, ICOS, Eurofleets, and JERICO suggests an interest in supporting semantic technologies and facilitating more structured and interoperable forms of data access.

Most of the RIs analyzed herein provide open access to their DOs, with only a few (e.g., ACTRIS, Eurofleets, and JERICO) requiring authentication for download, while all of them mandate authentication for uploading content (Figure 2D). Such access control mechanisms also help monitor how digital infrastructures and their DOs are used. This information is important for generating reliable key performance indicators, which are measurable metrics used to assess the efficiency of RIs in achieving their goals.^78^

The long-term accessibility of metadata is not always guaranteed, with only half of the analyzed RIs having metadata longevity plans in place (Figure 2F). Overall, the institutional commitment to metadata preservation is supported by the fact that all the RIs are sustained in the long term by the European Commission and/or by national funding resources. In most cases, their facilities are physically hosted by publicly funded institutes, ensuring their continued long-term operation.^79^ The existence of stable funding or institutional structures is a valuable foundation, but effective preservation requires dedicated strategies, resources, and governance actions. Additionally, without cohesive and clearly defined long-term financial plans, metadata preservation efforts remain inadequate. This concern about the lack of long-term commitment is supported by a recent review on how institutional repositories implement digital preservation policies, which highlights the limited number of published studies on this topic. The study suggested that repository managers prioritize other concerns over ensuring the long-term accessibility of their digital resources.^80^

### Interoperability

The metadata formats identified in our analysis largely reflect the prevailing standards widely adopted within the Semantic Web^81^^,^^82^ (Figure 3A). These formats provide structured and interoperable methods for managing metadata, ensuring consistency and ease of integration.^83^^,^^84^ Regarding data formats, the considerable convergence observed in the marine subdomain (i.e., NetCDF; Figure 3B) reflects the progress made in recent years in the field of interoperability within this area.^85^ Such progress, however, mainly concerns a small fraction of the data collected in the world’s oceans, including physical and meteorological variables, which are crucial for the operational oceanographic community.^86^ On the other hand, the biosphere subdomain requires greater and sustained efforts to satisfy data interoperability. A primary obstacle to achieving data interoperability in the biosphere subdomain is the heterogeneity of its data sources (e.g., remote sensing, traditional biotic surveying practices, metagenomics, and long-term observational data from monitoring programs).^87^ This diversity poses significant challenges for data integration tasks because data heterogeneity and interoperability issues are the primary barriers to effectively converting scientific data into meaningful information and knowledge.^88^^,^^89^ To overcome these obstacles, initiatives such as the Interoperable Descriptions of Observable Property Terminology (I-ADOPT) are essential. The I-ADOPT framework provides a structured ontology designed to harmonize variable descriptions across different communities, thereby facilitating the integration of heterogeneous ecological data and enhancing data interoperability.^90^

Nearly all the RIs analyzed herein make use of SAs (Figure 3C). In recent years, the field of environmental sciences has experienced significant growth in the use and publication of novel SAs.^54^ These standards are essential for representing and organizing the real world, and in some instances, they also enable the extraction and integration of information from (meta)data using computational techniques.^91^ Therefore, understanding the types of SAs that are in use within different RIs can provide useful information on how (meta)data are managed and how they can be queried and retrieved. For example, loose or semi-loose sets of terms such as glossaries, gazetteers, or thesauri are mostly used to annotate (meta)data and to precisely describe them.^92^ Conversely, ontologies can be used to model entities and any existing relationships among them, and they are valuable tools for representing complex systems and their connections. From the point of view of data management capacity, ontologies enable (meta)data integration, analysis, and inference processes through structured queries.^93^ The choice to use ontologies within RI information systems could provide indications of data management and interoperability practices.

### Reusability

Considerable variability was observed in the specific licenses and their applications (Figures 3D and 3E). This diversity arises from the coexistence of license types suited to specific DO categories (e.g., BSD, GNU GPL, GNU AGPL, and EUPL licenses for software) as well as from the diverse legal and institutional frameworks under which each RI operates. It should not be overlooked that subprinciple R1.1 emphasizes the need for both data and their associated metadata to be released with a clear usage license.^7^ Among the analyzed RIs, this was not always clear, especially for metadata. Notably, since the RIs are mainly funded by European Commission projects, it should be expected that at least their metadata are licensed under CC0 or equivalent public licenses.^94^

**Figure 4:**
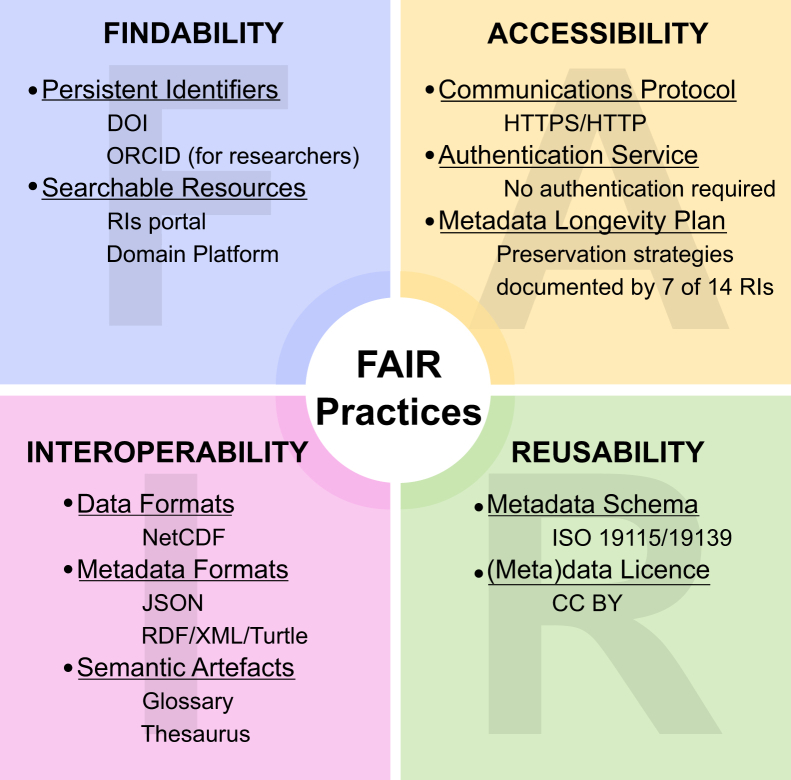
Summary of the main FAIR implementation practices adopted by the 14 analyzed research infrastructures participating in the ITINERIS project

Overall, the analysis presented here serves as a valuable reference point, as it marks the first detailed examination of the FAIR strategies adopted by RIs in the environmental domain at the Italian level. The resulting overview has laid the foundation for one of the main results of the ITINERIS project. In fact, this work provides essential insights for the design and development of the ITINERIS HUB, ensuring that the management of research outputs adheres to the FAIR principles. This work also sheds light on the FAIR implementation choices within specific subdomains, which are essential for the development of new platforms, digital archives, or new management systems and their compliance with FAIR strategies. Conducting such analyses is crucial for guiding future efforts, as informed decision-making in the early stages of platform development can significantly impact overall success. With this in mind, a condensed graphical overview is provided, summarizing the most common FAIR implementation practices that emerged from this analysis in the environmental domain (Figure 4).

### FAIR challenges in environmental science

The FAIR implementation approaches used for managing DOs vary widely across the analyzed environmental RIs, reflecting substantial heterogeneity among the current practices. As our results suggest, RIs usually implement FAIR practices that address the concrete needs and expectations of their scientific communities (e.g., the choice of the Ecological Metadata Language [EML] metadata schema for describing ecological data). However, this heterogeneity is also explained by the diverse nature of the DOs managed by the environmental RIs and considered in this study (i.e., research data, SAs, research codes, software, policy documents, training materials, and metadata records). Furthermore, FAIR practice diversity was also noted when the management of the same type of DO was considered, such as in the case of research data, which mirrors the intrinsic variability of environmental subdomains.^95^^,^^96^^,^^97^ The diversity of environmental digital resources poses a considerable challenge to convergence toward shared FAIR implementation solutions,^98^ and community-driven efforts should focus on aligning FAIR practices with the existing frameworks rather than reinventing the wheel, particularly for RIs that are managing similar types of DOs.^90^ Diverse FAIR implementation practices already exist because of past uncoordinated efforts to align with the FAIR principles.^15^ These efforts resulted in a variety of implementation practices tailored to specific community needs, which have generated a complex, and mostly disjoint, set of practices.^99^ While redundancies and/or overlaps exist, ensuring that they are avoided in the future to facilitate interoperability and reduce unnecessary complexity is essential. In fact, as the number of distinct practices increases, so does the effort required to ensure their interoperability, since alignments are needed. For example, within the EU-funded FAIRCORE4EOSC project, a Registry for Metadata Schema and Crosswalks (MSCR) has been developed that allows registered users and communities to create, register, and version schemas and crosswalks with PIDs. The MSCR also facilitates the transfer of (meta)data from one schema to another via registered crosswalks, thus enhancing the interoperability between information systems using different schemas.^100^ In addition to community-driven efforts, investments in informatics tools capable of mapping machine-readable schemas are pivotal for overcoming these challenges, as suggested by Kibria et al.^101^ and Magagna et al.^90^

Considering RI ecosystems more broadly, the factors influencing FAIR implementation choices are likely to result from a combination of disciplinary needs, technological maturity, and funding frameworks. One factor influencing the implementation pathways is the timing of adoption. The point in time at which an RI begins its journey toward FAIR practices significantly influences the adopted solutions. Earlier adopters often relied on technologies (and standards) that were available at the time, whereas more recently established RIs can benefit from access to updated tools, frameworks, and guidelines aligned with the FAIR principles. The availability and continuity of funding represent an additional factor influencing the choice of a FAIR solution. For example, RIs that manage their digital components at the national level often rely on project-based funding to develop and maintain their services. As a result, their capacity to adopt or expand FAIR practices may depend heavily on the success rate in competitive funding calls. In general, a critical observation emerging from our analysis is the lack of documentation regarding the motivations behind specific implementation choices. In many cases, it is difficult to trace why a particular FAIR solution was adopted or why one practice was preferred over another. This limits the possibility of learning from past decisions and sharing implementation rationales across different RIs. Thus, we encourage a more systematic and transparent approach to FAIR decision-making (including a preliminary assessment of the available practices and a clear justification of the selected solutions) as a way to support the development of coherent and reusable implementation strategies across RIs. An additional consideration is that the implementation of FAIR principles is still a relatively new process. Since their introduction in 2016, both technological enablers and community interpretations have continued to mature.^15^^,^^17^^,^^18^^,^^98^ The current implementation variability should therefore be viewed not only as the outcome of contextual constraints but also as part of an ongoing process of consolidation and refinement.

As a final message, we recommend that, whenever possible, shared and common practices for the implementation of FAIR principles should be adopted by the scientific community within the environmental domain. In fact, interoperability can be achieved only if there is convergence among the chosen FAIR practices. Therefore, the key recommendation is to reuse existing solutions whenever feasible. To ensure that this process is effectively realized, it is advisable that consortia or initiatives leading FAIR implementation efforts (such as the Research Data Alliance, the Go FAIR Foundation, and FAIR Connect) guide and facilitate this desired convergence toward established FAIR practices.^102^^,^^103^^,^^104^

From this perspective, the experience of the ITINERIS project has been extremely significant. At the national level, it marked the first time that scientific communities leading environmental RIs came together to create a single access point for the digital resources they manage (i.e., the ITINERIS HUB). This collaborative effort was based on several discussions and consultations regarding the FAIR implementation choices to be made, and this analysis was a fundamental part of the process. The ITINERIS project stands as an example of national coordination aimed at fostering the convergence of FAIR practices, an effort that we hope can support other scientific communities, in Europe and beyond, that will face the same challenges in the near future. Further steps on this topic will involve exploring the use of structured tools (i.e., the FAIR implementation profile), which are currently used within the Go FAIR community,^105^ for monitoring the technological choices implemented as collective decisions by RIs, their availability to scientific communities, and their convergence with recognized FAIR practices.^98^

It is also crucial to remember that behind RIs, platforms, tools, and services, there are individual technologists, researchers, IT specialists, and data stewards who generate, manage, and curate digital resources. These professionals make daily decisions on how to implement FAIR principles. For this reason, international, national, and institutional funding must support the continuous training and education of professionals in the application of FAIR solutions across different communities.^106^ In particular, practical training opportunities are key to guiding researchers in the adoption of best FAIR practices within their communities.^99^ Only through such a strategic vision can these experts serve as the driving force toward the desired convergence of FAIR implementation practices.

## Methods

A mixed-methods approach was used to provide an overview of the FAIR practices adopted by the RIs in the ITINERIS project. The 22 RIs participating in the ITINERIS project are ACTRIS ERIC, AnaEE ERIC, ATLaS, CeTrA, DANUBIUS-RI ERIC, DiSSCo, ECORD, eLTER, EMPHASIS, EMSO ERIC, EUFAR, Euro-Argo ERIC, Eurofleets, Geosciences IR, IBISBA, ICOS ERIC, JERICO, LifeWatch ERIC, LNS, R/V Laura Bassi, SIOS, and SMINO (Box 1). The mixed-methods approach, which integrates both qualitative and quantitative components, consisted of three consecutive and complementary steps: (1) an online survey, (2) one-on-one online interviews, and (3) an in-depth analysis of the acquired documentation and digital platforms. Step (1) involved all 22 RIs participating in the ITINERIS project, step (2) was carried out with 19 RIs (out of the 22 that were invited), while step (3) focused only on the 14 RIs with a minimum FAIR readiness level. By “minimum FAIR readiness level,” we refer to RIs that showed evidence of implementing FAIR practices in terms of at least three of the four FAIR principles. The entire data collection process and subsequent analysis took place between March 2023 and March 2024, and all the findings presented in this study refer to that period.

Box 1 **Research infrastructure abbreviations**

**ACTRIS ERIC**: Aerosol, Cloud, and Trace Gases Research Infrastructure
**AnaEE ERIC**: Analysis and Experimentation on Ecosystems
**ATLaS**: Advanced Technologies for LandSlides
**CeTrA**: Centre for Trace Analysis
**DANUBIUS-RI ERIC**: The International Centre for Advanced Studies on River-Sea Systems
**DiSSCo**: Distributed System of Scientific Collections
**ECORD**: European Consortium for Ocean Research Drilling
**eLTER**: Integrated European Long-Term Ecosystem, Critical Zone and Socio-ecological Research infrastructure
**EMPHASIS**: European Infrastructure for Multiscale Plant Phenomics and Simulation
**EMSO ERIC**: European Multidisciplinary Seafloor and Water Column Observatory
**EUFAR**: European Facility for Airborne Research
**Euro-Argo ERIC**: European contribution to the International Argo Programme
**Eurofleets**: Alliance of European marine research infrastructure for meeting the evolving needs of the research and industrial communities
**GeoSciences IR**: A research infrastructure for the Italian Geological Surveys Network
**IBISBA**: European Industrial Biotechnology Innovation and Synthetic Biology Accelerator
**ICOS ERIC**: Integrated Carbon Observation System
**JERICO**: Joint Pan-European Research Infrastructure for Coastal Observations
**LifeWatch ERIC**: e-Science European Infrastructure for Biodiversity and Ecosystem Research
**LNS**: Laboratori Nazionali del Sud
**R/V Laura Bassi:** Research Vessel Laura Bassi
**SIOS**: Svalbard Integrated Arctic Earth Observing System
**SMINO**: Northeast Italy monitoring system

List of acronyms and full names of the research infrastructures (RIs) participating in the ITINERIS project. ERIC stands for the European Research Infrastructure Consortium, the legal framework promoted by the European Commission to support the development and operation of RIs.

It is important to clarify two aspects of our methodology: first, the target of our analysis, and second, what fell within the spectrum of our analysis. RIs differ in their governance, management, and aims. Therefore, each RI has its own structure and function. This study, which has national coverage, focused on the Italian node of the RIs and their associated digital services where applicable (e.g., LifeWatch Italy). When a national node operated centralized digital services, we analyzed the services it provided or managed (e.g., ICOS ERIC and ACTRIS ERIC). When digital services or resources were not provided or managed at the national level, those available at the European level were considered (e.g., JERICO).

With respect to the “what,” our analysis focused on the following specific resources: research data, SAs, research codes, software, policy documents, training materials, and metadata records that RIs generate and/or manage. In this document, we use the term and definition of a DO when we refer to all these resources. The definition proposed by Kahn and Wilensky^107^ was adopted, whereby a DO is defined as “a sequence of bits, or a set of sequences of bits, incorporating a work or portion of a work or other information in which a party has rights or interests, or in which there is value, each of the sequences being structured in a way that is interpretable by one or more of the computational facilities and having as an essential element an associated unique persistent identifier.”

### Mixed-methods approach

To develop a comprehensive overview of the adoption of FAIR practices, we followed a mixed-methods approach, which consisted of three complementary data collection and analysis phases.

(1) Online survey submitted to RI representatives

The survey was submitted to the designated FAIR representative of each RI participating in the ITINERIS project for a total of 22 invitations. It was delivered as a spreadsheet file via a dedicated shared drive and consisted of 12 questions, with additional fields for the RI name and optional notes. Among the 12 questions, 8 were open ended and required the respondent to enter information such as links to the relevant resources, whereas the remaining 4 were multiple-choice questions, with predefined options that were selectable via drop-down menus.^108^ The questions were organized into three thematic sections: one focused on data and metadata, one on semantic resources, and one on other DOs produced or made available by each RI (e.g., research codes, software, policy documents, and training materials). This phase served as an exploratory step to gather preliminary insights into the state of FAIR practices across all RIs. A total of 22 survey responses were collected. In most cases, complete responses were obtained, although some entries included missing or unclear information, which required further clarification.^108^

(2) One-to-one online interviews

To verify and, where necessary, clarify and supplement the survey responses, individual consultations were proposed to all 22 RIs. Nineteen FAIR representatives participated, while three did not respond to the invitation. These meetings allowed (i) the clarification of misunderstood or misinterpreted questions; (ii) the correction or replacement of outdated, incorrect, or incomplete links and information; and (iii) the collection of additional information on specific FAIR practices that were not originally covered in the survey (i.e., PIDs, metadata schemas, and licenses). The interview process played a crucial role in further exploring initial responses and addressing any remaining information gaps. Based on the information collected in steps (1) and (2), only 14 RIs were subsequently analyzed in step (3). This decision stemmed from the observation that some RIs were still in the early stages of their construction processes and had therefore not yet implemented sufficiently established FAIR practices to allow for an in-depth analysis. As mentioned earlier, step (3) focused on the RIs with a minimum FAIR readiness level, which were defined as RIs evidencing FAIR implementation across at least three of the four principles.

(3) In-depth analysis of the documentation and digital platforms

**Figure 5:**
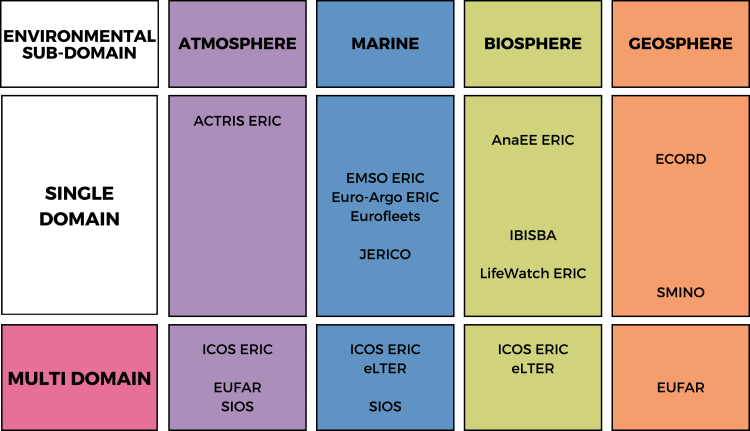
Classification of the 14 analyzed environmental research infrastructures across subdomains The 14 analyzed research infrastructures (RIs) were collocated within the four environmental subdomains (the atmosphere, marine, biosphere, and geosphere subdomains) and the multidomain category. The RIs were reported and classified according to the ESFRI Roadmap. Multidomain RIs were simultaneously reported in each subdomain of interest (e.g., ICOS was reported in the atmosphere, marine, and biosphere subdomains).

The final step involved a systematic review of the official documentation and digital platforms made available by the 14 RIs on their websites, portals, or catalogs.^33^ The 14 environmental RIs analyzed were ACTRIS ERIC, EMSO ERIC, Euro-Argo ERIC, Eurofleets, JERICO, AnaEE ERIC, IBISBA, LifeWatch ERIC, ECORD, SMINO, ICOS ERIC, eLTER, EUFAR, and SIOS, all of which are digital or hybrid. The RIs were classified into the atmosphere, marine, biosphere, and geosphere subdomains following the categorization scheme established by the ITINERIS project, which aligned with the ESFRI Roadmap^109^ (Figure 5). In cases where RIs spanned multiple domains, they were also classified under the “multidomain” category (Figure 5). The review of the official documentation and digital platforms was aimed at analyzing the FAIR practices presented in Figure 1. This process followed a stepwise strategy. Priority was given to institutional documents concerning DMPs and internal technical documentation. When information was not clearly documented, a direct verification was conducted. For instance, the (meta)data formats were checked by downloading and inspecting records, and the accessibility of data was verified to assess whether datasets were openly accessible or required authentication. The gathered information was subsequently validated by representatives of the RIs.

All the collected data were harmonized and organized into a structured spreadsheet to guarantee semantic consistency. For example, different variants (e.g., versions) referring to the same metadata standard were normalized under a single terminology (e.g., “NetCDF CF” vs. “NetCDF CF1.7”). This step was crucial for defining the categories that would later be used in the visual representations. Moreover, this harmonization process enabled the creation of a dataset^33^ that listed all identified practices and included direct links to each referenced resource.

The results obtained from the analysis were visualized through a series of Sankey diagrams. Specifically, diagrams illustrating the FAIR practices adopted by each infrastructure or group of infrastructures within each domain were created with R software (version 4.5.0) and the networkD3 package^110^ (version 0.4.1). This approach provided a concise overview of the different choices made and the convergence toward the most widely adopted practices.

## Resource availability

### Lead contact

Requests for additional information or resources should be directed to the lead contact, Dr. Enrica Nestola (enrica.nestola@cnr.it), who will ensure that these are provided.

### Materials availability

This study did not generate new materials.

### Data and code availability

The data used in this study are available at the Open Science Framework (OSF).
The dataset “Survey on FAIR Practices in Environmental Research Infrastructures” contains the questionnaire and the corresponding answers used to collect information on the digital resources managed by the environmental RIs involved in the ITINERIS project. It is available at https://doi.org/10.17605/OSF.IO/YNRPV.
The dataset “Identification of FAIR Practices in Environmental Research Infrastructures” presents the FAIR practices adopted by 14 environmental RIs involved in the ITINERIS project, along with references to the sources and documentation supporting each practice. It is based on an in-depth analysis of the official documentation and online resources available for the 14 environmental RIs examined. It is available at https://doi.org/10.17605/OSF.IO/PMG26.
The code used to create Figures 2 and 3 is available upon request.

## Acknowledgments

We would like to thank the FAIR representatives of the RIs who participated in the initial survey, providing an overview of the digital resources managed by their respective RIs, and who made themselves available for one-to-one meetings.

This study has been funded by EU – Next Generation EU Mission 4 “Education and Research”– component 2: “From research to business” – investment 3.1, “Fund for the realisation of an integrated system of research and innovation infrastructures”– project IR0000032 – ITINERIS – Italian Integrated Environmental Research Infrastructures System– CUP B53C22002150006. The authors acknowledge the research infrastructures participating in the ITINERIS project with their Italian nodes: ACTRIS, AnaEE, ATLaS, CeTrA, DANUBIUS, DiSSCo, e-LTER, ECORD, EMPHASIS, EMSO, EUFAR, Euro-Argo, Eurofleets, Geoscience, IBISBA, ICOS, JERICO, LifeWatch, LNS, N/R Laura Bassi, SIOS, and SMINO. Views and opinions expressed are however those of the author(s) only and do not necessarily reflect those of the European Union or the European Commission. Neither the European Union nor European Commission can be held responsible for them.

## Author contributions

I.R. conceived the study. E.N. designed the manuscript structure and coordinated the overall writing process. E.N., G.S., G.I., and A.T. performed the data collection process and analyzed the results. E.N. and G.S. wrote the first draft of the manuscript. G.I. developed the code for the Sankey diagrams and provided Figures 2 and 3. G.S. and A.N.M. developed Figures 1, 4, and 5 and the graphical abstract. D.R. supported the writing of the accessibility section. C.D.M. and A.N.M. supported the writing of the interoperability section. All the authors critically reviewed the manuscript.

## Declaration of interests

The authors declare that they have no competing interests.

## Declaration of generative AI and AI-assisted technologies in the writing process

During the preparation of this work, the authors used ChatGPT to improve the clarity, readability, and English grammar of the work. After using this tool, the authors reviewed and edited the content as needed and take full responsibility for the content of the publication.

## References

[1] Nault R., Cave M.C., Ludewig G., Moseley H.N.B., Pennell K.G., Zacharewski T. (2023). A Case for Accelerating Standards to Achieve the FAIR Principles of Environmental Health Research Experimental Data. Environ. Health Perspect..

[2] Bumberger J., Abbrent M., Brinckmann N., Hemmen J., Kunkel R., Lorenz C., Lünenschloss P., Palm B., Schnicke T., Schulz C. (2025). Digital ecosystem for FAIR time series data management in environmental system science. SoftwareX.

[3] Dumit V.I., Ammar A., Bakker M.I., Bañares M.A., Bossa C., Costa A., Cowie H., Drobne D., Exner T.E., Farcal L. (2023). From principles to reality. FAIR implementation in the nanosafety community. Nano Today.

[4] European Commission, Directorate-General for Research & Innovation (2016). H2020 Programme: Guidelines on FAIR Data Management in Horizon 2020 Version 3.0. https://ec.europa.eu/research/participants/data/ref/h2020/grants_manual/hi/oa_pilot/h2020-hi-oa-data-mgt_en.pdf.

[5] Burgelman J.-C., Pascu C., Szkuta K., Von Schomberg R., Karalopoulos A., Repanas K., Schouppe M. (2019). Open Science, Open Data, and Open Scholarship: European Policies to Make Science Fit for the Twenty-First Century. Front. Big Data.

[6] Kumar P., Hendriks T., Panoutsopoulos H., Brewster C. (2024). Investigating FAIR data principles compliance in horizon 2020 funded Agri-food and rural development multi-actor projects. Agric. Syst..

[7] Wilkinson M.D., Dumontier M., Aalbersberg I.J., Appleton G., Axton M., Baak A., Blomberg N., Boiten J.-W., da Silva Santos L.B., Bourne P.E. (2016). The FAIR Guiding Principles for scientific data management and stewardship. Sci. Data.

[8] Schultes E. (2023). FAIR digital objects for academic publishers. Inf. Serv. Use.

[9] Petzold A., Asmi A., Vermeulen A., Pappalardo G., Bailo D., Schaap D., Glaves H.M., Bundke U., Zhao Z. (2019). 2019 15th International Conference on eScience (eScience).

[10] Schultes E., Strawn G., Mons B. (2018). Proceedings of the XX International Conference “Data Analytics and Management in Data Intensive Domains” (DAMDID/RCDL’2018).

[11] Dillo, I., Hodson, S., Pittonet Gaiarin, S., and Grootveld, M. (2021). D5.7 Recommendations for a FAIR EOSC - White Paper FAIRsFAIR Synchronisation Force 2021. Zenodo. https://zenodo.org/records/5744786.

[12] Parland-von Essen J., Dillo I. (2022). FAIR-IMPACT. Res. Ideas Outcomes.

[13] Bundke U., Bailo D., Carval T., Cervone L., De Nart D., Dema C., Ferrari T., Petzold A., Thijsse P., Vermeulen A., Zhao Z. (2024). EGU General Assembly 2024, Vienna, Austria, 14–19 Apr 2024, EGU24-8465.

[14] Guerrero C.U., Romero M.V., Dolman M., Dumontier M. (2023). FAIR Begins at home: Implementing FAIR via the Community Data Driven Insights. arXiv.

[15] Jacobsen A., De Miranda Azevedo R., Juty N., Batista D., Coles S., Cornet R., Courtot M., Crosas M., Dumontier M., Evelo C.T. (2020). FAIR Principles: Interpretations and Implementation Considerations. Data Intell..

[16] Wittenburg P., de Jong F., van Uytvanck D., Cocco M., Jeffery K., Lautenschlager M., Thiemann H., Hellström M., Asmi A., Holub P. (2020). State of FAIRness in ESFRI Projects. Data Intell..

[17] van Reisen M., Stokmans M., Basajja M., Ong’ayo A.O., Kirkpatrick C., Mons B. (2020). Towards the Tipping Point for FAIR Implementation. Data Intell..

[18] Mons B., Schultes E., Liu F., Jacobsen A. (2020). The FAIR Principles: First Generation Implementation Choices and Challenges. Data Intell..

[19] Pujol Priego L., Wareham J., Romasanta A.K.S. (2022). The puzzle of sharing scientific data. Ind. Innovat..

[20] Lannom L., Koureas D., Hardisty A.R. (2020). FAIR Data and Services in Biodiversity Science and Geoscience. Data Intell..

[21] Amaro R.E., Åqvist J., Bahar I., Battistini F., Bellaiche A., Beltran D., Biggin P.C., Bonomi M., Bowman G.R., Bryce R.A. (2025). The need to implement FAIR principles in biomolecular simulations. Nat. Methods.

[22] Koers H., Bangert D., Hermans E., van Horik R., de Jong M., Mokrane M. (2020). Recommendations for Services in a FAIR Data Ecosystem. Patterns.

[23] Bailo D., Paciello R., Michalek J., Cocco M., Freda C., Jeffery K., Atakan K. (2023). The EPOS multi-disciplinary Data Portal for integrated access to solid Earth science datasets. Sci. Data.

[24] European Commission (2021). EU Regulation 2021/695. https://eur-lex.europa.eu/eli/reg/2021/695/oj.

[25] OECD (2019). Reference Framework for Assessing the Scientific and Socio-Economic Impact of Research Infrastructures. OECD Science, Technology and Industry Policy Papers https://www.oecd.org/en/publications/reference-framework-for-assessing-the-scientific-and-socio-economic-impact-of-research-infrastructures_3ffee43b-en.html.

[26] Bolliger I.K., Griffiths A. (2020). Big Science and Research Infrastructures in Europe.

[27] European Strategy Forum on Research Infrastructures. ESFRI-EOSC Coordination Task Force. https://www.esfri.eu/working-groups/esfri-eosc-task-force.

[28] David R., Rybina A., Burel J.M., Heriche J.K., Audergon P., Boiten J.W., Coppens F., Crockett S., Exter K., Fahrner S. (2023). “Be sustainable”: EOSC-Life recommendations for implementation of FAIR principles in life science data handling. EMBO J..

[29] Petzold, A., Hienola, A., Ewbank, J., Tedds, J., Lamanna, G., Bird, I., Gotz, A., Bodera, J., de Jong, F., and Wolff-Boenisch, B. (2024). Science Clusters: Position statement on operational commitment to EOSC and Open Research. Zenodo. 10.5281/zenodo.10732048.

[30] Petzold, A., Blomberg, N., Lamanna, G., Dimper, R., and Dekker, R. (2021). ESFRI Science Clusters: Position Statement on Expectations and Long-Term Commitment in Open Science. Zenodo. 10.5281/zenodo.4892245.

[31] Cornacchia, C., and Rosati, I. (2023). Access to facilities, FAIR data and related services: the ITINERIS project, EGU General Assembly 2023, Vienna, Austria, 24–28 Apr 2023, EGU23-15909, 10.5194/egusphere-egu23-15909.

[32] Dema C., Izzi F., Mona L., Salvia V., Cornacchia C., Ripepi E., Volini M., Pappalardo G. (2024). EGU General Assembly 2024, Vienna, Austria, 14–19 Apr 2024, EGU24-19603.

[33] Nestola E., Ingrosso G., Tarallo A., Sgrigna G., Di Muri C., Muresan A., Raho D., Rosati I. (2025). Identification of FAIR practices in environmental Research Infrastructures. Open Science Framework.

[34] Hellström M., Johnsson M., Vermeulen A., Zhao Z., Hellström M. (2020). Towards Interoperable Research Infrastructures for Environmental and Earth Sciences: A Reference Model Guided Approach for Common Challenges.

[35] de Castro, P., Herb, U., Rothfritz, L., and Schöpfel, J. (2023). Building The Plane as We Fly It: The Promise of Persistent Identifiers. Zenodo. 10.5281/zenodo.7258286.

[36] McMurry J.A., Juty N., Blomberg N., Burdett T., Conlin T., Conte N., Courtot M., Deck J., Dumontier M., Fellows D.K. (2017). Identifiers for the 21st century: How to design, provision, and reuse persistent identifiers to maximize utility and impact of life science data. PLoS Biol..

[37] Xie I., Matusiak K.K., Xie I., Matusiak K.K. (2016). Discover Digital Libraries.

[38] Dutta B., Toulet A., Emonet V., Jonquet C., Garoufallou E., Virkus S., Siatri R., Koutsomiha D. (2017). Metadata and Semantic Research.

[39] ORCID Record Schema ORCID. https://info.orcid.org/documentation/integration-guide/orcid-record/.

[40] European Commission (2016). DCAT Application Profile for data portals in Europe. https://op.europa.eu/en/web/eu-vocabularies/dcat-ap.

[41] Weigel T., Schwardmann U., Klump J., Bendoukha S., Quick R. (2020). Making Data and Workflows Findable for Machines. Data Intell..

[42] Boeckhout M., Zielhuis G.A., Bredenoord A.L. (2018). The FAIR guiding principles for data stewardship: fair enough?. Eur. J. Hum. Genet..

[43] Nost E., Gehrke G., Vera L., Hansen S. (2025). Why the Environmental Data & Governance Initiative is archiving public environmental data. Patterns.

[44] Tanhua T., Pouliquen S., Hausman J., O’Brien K., Bricher P., de Bruin T., Buck J.J.H., Burger E.F., Carval T., Casey K.S. (2019). Ocean FAIR Data Services. Front. Mar. Sci..

[45] PubNub glossary (2024). What is communication protocol? PubNub. https://www.pubnub.com/learn/glossary/communication-protocols/.

[46] He Z., Tang W., Yang X., Wang L., Liu J. (2014). Use of NTRIP for Optimizing the Decoding Algorithm for Real-Time Data Streams. Sensors.

[47] Hou L., Zhao S., Li X., Chatzimisios P., Zheng K. (2017). Design and implementation of application programming interface for Internet of things cloud. Int. J. Network Mgmt..

[48] Hagedorn P., Liu L., König M., Hajdin R., Blumenfeld T., Stöckner M., Billmaier M., Grossauer K., Gavin K. (2023). BIM-Enabled Infrastructure Asset Management Using Information Containers and Semantic Web. J. Comput. Civ. Eng..

[49] Epping M., Morowczynski M. (2021). Authentication and Authorization (v2). IDPro Body of Knowledge.

[50] Landi A., Thompson M., Giannuzzi V., Bonifazi F., Labastida I., da Silva Santos L.O.B., Roos M. (2020). The “A” of FAIR – As Open as Possible, as Closed as Necessary. Data Intell..

[51] Li C., Sugimoto S. (2017). Provenance Description of Metadata Vocabularies for the Long-term Maintenance of Metadata. J. Data Inf. Sci..

[52] Ahmad R., Rafiq M. (2023). Global perspective on digital preservation policy: A systematic review. J. Librarian. Inf. Sci..

[53] De Santis L. (2024). FAIR as a Journey: Lessons Learned from Building the GoTriple Discovery Platform for Social Sciences and Humanities. Publications.

[54] Di Muri C., Pulieri M., Raho D., Muresan A.N., Tarallo A., Titocci J., Nestola E., Basset A., Mazzoni S., Rosati I. (2024). Assessing semantic interoperability in environmental sciences: variety of approaches and semantic artefacts. Sci. Data.

[55] Le Franc, Y., Parland-von Essen, J., Bonino, L., Lehväslaiho, H., Coen, G., and Staiger, C. (2020). D2.2 FAIR Semantics: First recommendations. Zenodo. 10.5281/zenodo.3707984.

[56] British Oceanographic Data Centre (2024). The NERC Vocabulary Server, Natural Environment Research Council. https://vocab.nerc.ac.uk/.

[57] Vrousgou O., Burdett T., Parkinson H., Jupp S., Stefanidis K. (2016). Proceedings of the Workshops of the EDBT/ICDT Joint Conference 2016.

[58] Tarallo A., Pulieri M., Ramezani P., Rosati I. (2024). Advancements in EcoPortal: Enhancing functionalities for the ecological domain semantic artefacts repository. FAIR Connect.

[59] Labastida I., Margoni T. (2020). Licensing FAIR Data for Reuse. Data Intell..

[60] Fernández-López M., Poveda-Villalón M., Suárez-Figueroa M.C., Gómez-Pérez A. (2019). Why are ontologies not reused across the same domain?. J. Web Semantics.

[61] Manso-Narvarte I., Solabarrieta L., Caballero A., Anabitarte A., Knockaert C., Dhondt C.A.L., Fernandes-Salvador J.A. (2024). Fishing vessels as met-ocean data collection platforms: data lifecycle from acquisition to sharing. Front. Mar. Sci..

[62] Zulfiqar M., Crusoe M.R., König-Ries B., Steinbeck C., Peters K., Gadelha L. (2024). Implementation of FAIR Practices in Computational Metabolomics Workflows—A Case Study. Metabolites.

[63] Peterseil J., Offenthaler I., Wohner C., Magagna B., Schultes E., Lund Myhre C., Jeffery K., Bailo D., Dobler D., Portier M. (2023). ENVRI-FAIR D5.6: Synthesis and future strategy. Zenodo.

[64] The DOI Foundation https://www.doi.org/the-foundation/about-us/.

[65] Klump J., Huber R. (2017). 20 Years of Persistent Identifiers – Which Systems are Here to Stay?. Data Sci. J..

[66] Brodeur J., Coetzee S., Danko D., Garcia S., Hjelmager J. (2019). Geographic Information Metadata—An Outlook from the International Standardization Perspective. ISPRS Int. J. GeoInf..

[67] Hassell D., Gregory J., Blower J., Lawrence B.N., Taylor K.E. (2017). A data model of the Climate and Forecast metadata conventions (CF-1.6) with a software implementation (cf-python v2.1). Geosci. Model Dev. (GMD).

[68] Eggleton F., Winfield K. (2020). Open Data Challenges in Climate Science. Data Sci. J..

[69] Dietze M.C., Thomas R.Q., Peters J., Boettiger C., Koren G., Shiklomanov A.N., Ashander J. (2023). A community convention for ecological forecasting: Output files and metadata version 1.0. Ecosphere.

[70] Leipzig J., Nüst D., Hoyt C.T., Ram K., Greenberg J. (2021). The role of metadata in reproducible computational research. Patterns.

[71] Ulrich H., Kock-Schoppenhauer A.-K., Deppenwiese N., Gött R., Kern J., Lablans M., Majeed R.W., Stöhr M.R., Stausberg J., Varghese J. (2022). Understanding the Nature of Metadata: Systematic Review. J. Med. Internet Res..

[72] Lecarpentier D., Wittenburg P., Elbers W., Michelini A., Kanso R., Coveney P., Baxter R. (2013). EUDAT: A New Cross-Disciplinary Data Infrastructure for Science. Int. J. Digit. Curation.

[73] Tenopir C., Rice N.M., Allard S., Baird L., Borycz J., Christian L., Grant B., Olendorf R., Sandusky R.J. (2020). Data sharing, management, use, and reuse: Practices and perceptions of scientists worldwide. PLoS One.

[74] Rodriguez M. (2018). HTTPS Everywhere: Industry Trends and the Need for Encryption. Ser. Rev..

[75] Postel, J., and Reynolds, J. (1985). File Transfer Protocol (Internet Engineering Task Force) 10.17487/RFC0959.

[76] Springall, D., Durumeric, Z., and Halderman, J.A. (2016). FTP: The Forgotten Cloud. In 2016 46th Annual IEEE/IFIP International Conference on Dependable Systems and Networks (DSN), pp. 503–513. 10.1109/DSN.2016.52.

[77] Alkan R.M., Erol S., İlçi V., Ozulu İ.M. (2020). Comparative analysis of real-time kinematic and PPP techniques in dynamic environment. Measurement.

[78] ESFRI Working Group Report (2019). Monitoring of Research Infrastructures Performance. https://www.esfri.eu/sites/default/files/ESFRI_WG_Monitoring_Report.pdf.

[79] European Commission (2020). Supporting the Transformative Impact of Research Infrastructures on European Research. https://research-and-innovation.ec.europa.eu/knowledge-publications-tools-and-data/publications/all-publications/supporting-transformative-impact-research-infrastructures-european-research_en.

[80] Barrueco J.M., Termens M. (2022). Digital preservation in institutional repositories: a systematic literature review. Digit. Libr. Perspect..

[81] Decker S., Melnik S., van Harmelen F., Fensel D., Klein M., Broekstra J., Erdmann M., Horrocks I. (2000). The Semantic Web: the roles of XML and RDF. IEEE Internet Comput..

[82] Batista D., Gonzalez-Beltran A., Sansone S.-A., Rocca-Serra P. (2022). Machine actionable metadata models. Sci. Data.

[83] Chan L.M., Zeng M.L. (2006). Metadata Interoperability and Standardization - A Study of Methodology Part I: Achieving Interoperability at the Schema Level. D-Lib Mag..

[84] Musen M.A., O’Connor M.J., Schultes E., Martínez-Romero M., Hardi J., Graybeal J. (2022). Modeling community standards for metadata as templates makes data FAIR. Sci. Data.

[85] Snowden D., Tsontos V.M., Handegard N.O., Zarate M., O’ Brien K., Casey K.S., Smith N., Sagen H., Bailey K., Lewis M.N., Arms S.C. (2019). Data Interoperability Between Elements of the Global Ocean Observing System. Front. Mar. Sci..

[86] Alvarez Fanjul E., Ciliberti S., Pearlman J., Wilmer-Becker K., Bahurel P., Ardhuin F., Arnaud A., Azizzadenesheli K., Aznar R., Bell M. (2024). Promoting best practices in ocean forecasting through an Operational Readiness Level. Front. Mar. Sci..

[87] Wieczorek J., Bloom D., Guralnick R., Blum S., Döring M., Giovanni R., Robertson T., Vieglais D. (2012). Darwin Core: An Evolving Community-Developed Biodiversity Data Standard. PLoS One.

[88] Michener W.K., Allard S., Budden A., Cook R.B., Douglass K., Frame M., Kelling S., Koskela R., Tenopir C., Vieglais D.A. (2012). Participatory design of DataONE—Enabling cyberinfrastructure for the biological and environmental sciences. Ecol. Inform..

[89] Kissling W.D., Hardisty A., García E.A., Santamaria M., De Leo F., Pesole G., Freyhof J., Manset D., Wissel S., Konijn J., Los W. (2015). Towards global interoperability for supporting biodiversity research on essential biodiversity variables (EBVs). Biodiversity.

[90] Magagna B., Rosati I., Stoica M., Schindler S., Moncoiffe G., Devaraju A., Peterseil J., Huber R. (2021). The I-ADOPT Interoperability Framework for FAIRer data descriptions of biodiversity. arXiv.

[91] Diepenbroek M., Schindler U., Huber R., Pesant S., Stocker M., Felden J., Buss M., Weinrebe M. (2017). Terminology supported archiving and publication of environmental science data in PANGAEA. J. Biotechnol..

[92] Mazzocchi F. (2018). Knowledge Organization System (KOS): An Introductory Critical Account. KO.

[93] Cardoso de Oliveira C., Bragato Barros T.H. (2024). Ontologies and Research Data: A Theoretical and Methodological Overview. cjils-rcsib..

[94] European Commission (2025). AGA – Annotated Grant Agreement EU Funding Programmes 2021-2027. https://horizoneurope.apre.it/pubblicata-la-versione-1-0-dellaga-annotated-grant-agreement-eu-funding-programmes-2021-2027/.

[95] Darch P.T., Borgman C.L., Traweek S., Cummings R.L., Wallis J.C., Sands A.E. (2015). What lies beneath?: Knowledge infrastructures in the subseafloor biosphere and beyond. Int. J. Digit. Libr..

[96] Martin P., Chen Y., Hardisty A., Jeffery K., Zhao Z., Chabbi A., Loescher H.W. (2017). Terrestrial Ecosystem Research Infrastructures.

[97] Garcia-Silva A., Gomez-Perez J.M., Palma R., Krystek M., Mantovani S., Foglini F., Grande V., De Leo F., Salvi S., Trasatti E. (2019). Enabling FAIR research in Earth Science through research objects. Future Gener. Comput. Syst..

[98] Schultes E., Magagna B., Hettne K.M., Pergl R., Suchánek M., Kuhn T., Grossmann G., Ram S. (2020). Advances in Conceptual Modeling.

[99] Hughes L.D., Tsueng G., DiGiovanna J., Horvath T.D., Rasmussen L.V., Savidge T.C., Stoeger T., Turkarslan S., Wu Q., Wu C. (2023). Addressing barriers in FAIR data practices for biomedical data. Sci. Data.

[100] Suominen T., Kesäniemi J., Koivula H. (2023). Making Schemas and Mappings Available and FAIR: A metadata and schema crosswalk registry from the FAIRCORE4EOSC project. Biodivers. Inf. Sci. Stand..

[101] Kibria, M.G., Ali, S., Jarwar, M.A., and Chong, I. (2017). A framework to support data interoperability in web objects based IoT environments. In 2017 International Conference on Information and Communication Technology Convergence (ICTC), pp. 29–31. 10.1109/ICTC.2017.8190935.

[102] FAIR Connect https://fairconnect.pro/.

[103] GO FAIR Foundation. https://www.gofair.foundation.

[104] Research Data Alliance. https://www.rd-alliance.org/.

[105] GO FAIR initiative: Make your data & services FAIR GO FAIR. https://www.go-fair.org/.

[106] Shanahan H., Hoebelheinrich N., Whyte A. (2021). Progress toward a comprehensive teaching approach to the FAIR data principles. Patterns.

[107] Kahn R., Wilensky R. (2006). A framework for distributed digital object services. Int. J. Digit. Libr..

[108] Nestola, E., Ingrosso, G., Tarallo, A., Sgrigna, G., Muresan, A., Di Muri, C., Raho, D., and Rosati, I. (2025). Survey on FAIR Practices in environmental Research Infrastructures. Open Science Framework. 10.17605/OSF.IO/YNRPV. PMC12827747 41583974

[109] ESFRI (2021). Roadmap & Strategy Report on Research Infrastructures. 10.25607/OBP-1861.

[110] Allaire, J.J., Gandrud, C., Russell, K., and Yetman, C. (2014). networkD3: D3 JavaScript Network Graphs from R. (The Comprehensive R Archive Network). 10.32614/CRAN.package.networkD3.

